# Markers of Memory CD8 T Cells Depicting the Effect of the BNT162b2 mRNA COVID-19 Vaccine in Japan

**DOI:** 10.3389/fimmu.2022.836923

**Published:** 2022-04-28

**Authors:** Hiroyuki Kondo, Takahiro Kageyama, Shigeru Tanaka, Kunihiro Otsuka, Shin-ichi Tsukumo, Yoichi Mashimo, Yoshihiro Onouchi, Hiroshi Nakajima, Koji Yasutomo

**Affiliations:** ^1^ Department of Immunology & Parasitology, Graduate School of Medicine, Tokushima University, Tokushima, Japan; ^2^ Department of Allergy and Clinical Immunology, Graduate School of Medicine, Chiba University, Chiba, Japan; ^3^ Department of Public Health, Graduate School of Medicine, Chiba University, Chiba, Japan; ^4^ Department of Interdisciplinary Researches for Medicine and Photonics, Institute of Post-LED Photonics, Tokushima University, Tokushima, Japan; ^5^ The Research Cluster Program on Immunological Diseases, Tokushima University, Tokushima, Japan; ^6^ Tokushima International Science Institute (TISI), Tokushima University, Tokushima, Japan

**Keywords:** COVID-19, T cell, CD8, vaccine, markers, antigen

## Abstract

BNT162b2, a nucleoside-modified mRNA vaccine for SARS-CoV-2 spike glycoprotein (S), provides approximately 95% efficacy for preventing COVID-19. However, it remains unclear how effectively memory CD8+ T cells are generated and which genetic and environmental factors affect the generation and function of memory CD8+ T cells elicited by this vaccine. Here, we investigated the frequency and functions of memory CD8+ T cells 3 weeks after the second vaccination in the Japanese population. Using a peptide-MHC pentamer, we detected an increased number of memory CD8+ T cells together with increased serum anti-S protein antibody in females compared with that in males, but the frequency of pentamer-positive cells was not positively correlated with antibody titers. Memory precursor effector cells (KLRG1-CD127+) among both CD8+ cells and pentamer+ cells and effector cells (CD38-HLA-DR+) among pentamer+ cells were more abundant in females than in males. Upon S protein-mediated stimulation of T cells, the intensity of CD107a and granzyme B expression was increased in females compared with that in males, indicating stronger memory CD8+ T cell responses in females than in males. Our studies showed that the BNT162b2 vaccine elicits increased memory CD8+ T cell proliferation and secondary CTL responses in females compared with those in males in the Japanese population. These findings provide an important basis for the distinct sex difference in cellular immune responses to mRNA vaccination and suggest that memory precursor effector cells can be one of markers to evaluate and boost cellular immunity induced by BNT162b2.

## Introduction

Infection with severe acute respiratory syndrome coronavirus 2 (SARS-CoV-2) occurred in late 2019 and rapidly spread worldwide (1–3). The disease (coronavirus disease 2019, COVID-19) has caused significant morbidity and mortality, with the emergence of viruses with various mutations in the spike glycoprotein. Worldwide, various attempts have been applied to develop novel vaccines and therapeutic strategies together with assessing immune responses against SARS-CoV-2 (4, 5).

The S protein of SARS-CoV-2 is needed for the entry of virus into the cells through interaction with angiotensin-converting enzyme II (6). The S protein includes a signal peptide, an S1 protease fragment that contains a receptor-binding domain and an S2 protease fragment. By expecting the generation of memory T and B cells against the S protein, BioNTech and Pfizer launched a new type of mRNA vaccine that encodes the SARS-CoV-2 full-length spike with modification by two proline mutations. Clinical trials of the vaccine revealed approximately 95% efficacy for protecting against COVID-19 (7, 8). The vaccine increased the serum levels of neutralizing antibodies against the S protein and IFN-γ production from CD4+ and CD8+ T cells (9). The generation of T cell memory after vaccination is required for long-term protective immunity to SARS-CoV-2. Indeed, one paper showed that memory T cells contribute to protection against SARS-CoV-2 rechallenge in a rhesus macaque model (10). However, it remains unclear which environmental factors affect the generation of memory T cells, which are markers for memory T cells and how long protective immunity is maintained.

To this end, we tested the frequency and function of CD8+ T cell responses against the S protein of SARS-CoV-2 before and three weeks after the second dose of the BNT162b2 mRNA vaccine in the Japanese population. We found an increased number of memory CD8+ T cells in females compared with that in males, but the frequency of S protein-specific CD8+ T cells was not positively correlated with antibody titers. The numbers of total and antigen-specific memory precursor effector cells (KLRG1-CD127+CD8+) and antigen-specific effector cells (CD38-HLA-DR+CD8+) were increased in females compared those in with males. These findings provide an important basis for the distinct sex differences in the cellular immune responses to mRNA vaccines and suggest that memory precursor effector cells can serve as markers to boost vaccine effectiveness.

## Materials and Methods

### Patient Recruitment

Healthcare workers in Chiba University Hospital who received the BNT162b2 mRNA COVID-19 vaccine (Pfizer, Inc., and BioNTech) were recruited. Blood samples were collected 0–2 weeks before the 1^st^ dose and 3 weeks after the 2^nd^ dose. Antibody responses were analyzed using Elecsys^®^ Anti-SARS-CoV-2S on the Cobas 8000 e801 module (Roche Diagnostics, Rotkreuz, Switzerland). Background information was collected (11), and all participants gave written informed consent before undergoing any study procedures. This study was approved by the ethical committee for medical research involving human subjects at Chiba University and Tokushima University.

### PBMC Isolation

PMBCs were isolated from peripheral blood and stored in liquid nitrogen (11). Frozen PMBCs were transported from Chiba University to Tokushima University and stored in liquid nitrogen. Stored PMBCs were thawed and immediately used in various experiments.

### Antibody Titer

Serum was prepared by centrifugation of blood samples followed by storage at -80°C, with analysis of thawed samples. Antibody titer was measured as we have previously reported (11). Antibody titers were converted to log 2 values for presentation and statistical analysis. Antibody responses were analyzed using Elecsys^®^ Anti-SARS-CoV-2S on a Cobas 8000 e801 module (Roche Diagnostics, Rotkreuz, Switzerland). This system allows for the quantitative detection of antibodies, predominantly IgG, specific for the SARS-CoV-2 spike protein receptor-binding domain. Samples with a titer >250 U/mL were diluted until the titer became 250 U/mL according to the manufacturer’s protocol.

### HLA Screening

Genomic DNA was extracted from white blood cells and amplified with primers for HLA-A*02, forward primer 5’- CACTCCTCGTCCCCAGGCTGT -3’ and reverse primer 5’- CGTGGCCCCTGGTACCCGT-3’, and HLA-A*0201, forward primer 5’- TCCTCGTCCCCAGGCTCT -3’ and reverse primer 5’- GTGGCCCCTGGTACCCGT-3’, with a thermal cycler (TaKaRa). We confirmed the gene by DNA sequencing (Applied Biosystems 3130).

### Flow Cytometry

Cryopreserved PBMCs were thawed and stained with APC-labeled HLA-A*0201 pentamers (YLQPRTFLL: SARS-CoV-2 S_269_) and antibodies. For pentamer staining, the cells were incubated for 15 min at room temperature, washed, and then stained with fluorochrome-conjugated antibodies for specific surface markers for 20 min at 4°C. The antibody mixture consisted of anti-human CD3 (Biolegend, clone HIT3a), CD8α (Biolegend, clone HIT8a), CCR7 (Biolegend, clone G043H7), CD45RA (Biolegend, clone HI100), KLRG1 (Biolegend, clone SA231A2), CD127 (Biolegend, clone A019D5), CD28 (Biolegend, clone CD28.2), PD-1 (Biolegend, clone EH12.2H7), CD38 (Biolegend, clone HIT2), HLA-DR (Biolegend, clone L234), and CD107a (Biolegend, clone H4A3). After three additional washes with FACS buffer, the cells were analyzed on a FACSCanto II (BD Biosciences).

### T Cell Stimulation

Cryopreserved PBMCs (5×105 cells) were thawed and stimulated in the presence of a peptide (YLQPRTFLL, 10 μg/ml) and human IL-2 (20 ng/ml) for 14 days using a U-bottom 96-well plate.

### Cytokine Measurement

Cytokine concentrations were measured using the LEGENDplex™ Human CD8/NK Panel (13-plex) with a Filter Plate (Biolegend). In brief, we incubated cell culture supernatant with the human CD8/NK panel premixed beads on a plate shaker. After incubation, human CD8/NK panel detection antibodies were added to the mixed solution and incubated on a plate shaker. After incubation, the mixed solution was analyzed on a FACSCanto II (BD Biosciences).

### Statistical Analysis

The Mann–Whitney U test or Wilcoxon signed-rank test was used to compare data between two unpaired groups or between two paired groups, respectively. The Spearman correlation test was used to assess the significance of the correlation.

## Results

### Study Design

**Figure 1:**
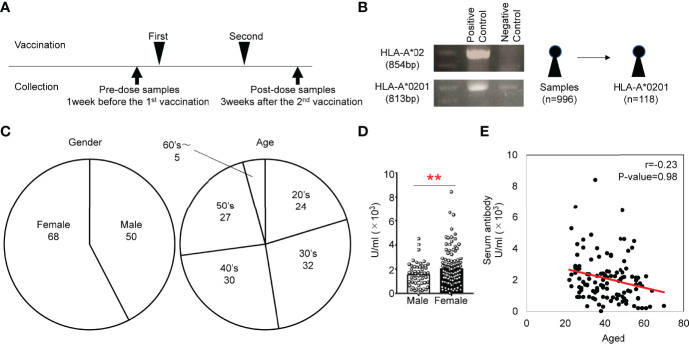
**(A)** Study scheme for the collection of PBMCs from individuals administered the BNT162b2 vaccine. **(B)** Genotyping PCR analysis of genomic DNA from human white blood cells. Gel electrophoresis of the amplified HLA-A*02 fragment (854 bp) and HLA-*0201 fragment (813 bp). **(C)** Frequencies of sex and age of subjects that provided HLA-A*0201^+^ samples. **(D)** Antibody titers of males and females in HLA-A*0201^+^ samples of after vaccination. **(E)** Scatter plots of antibody titer and cell number of HLA-A*0201^+^ samples after vaccination. **p < 0.01.

We recruited 996 donors at Chiba University who received 2 rounds of administration of BNT162b2, a nucleoside-modified mRNA vaccine (**Figure 1A**) (11). Approximately 10% of Japanese individuals carry HLA-A*0201 (12), and several epitopes of SARS-CoV-2 on HLA-A0201 have been reported (13). Thus, we first screened donors who carry HLA-A*0201 to detect S protein-specific T cells with pentamers composed of epitopes of SAR-CoV-2 and HLA-A0201 (**Figure 1B**). We amplified genomic DNA with specific primers for HLA-A*2 and A*0201 and found 118 donors carrying the HLA-A*0201 gene (**Figure 1B**). We confirmed the genotypes by DNA sequencing. The donors included 68 females and 50 males, and the ages ranged from 20 to 70 (**Figure 1C**). The antibody titer against the S-protein was higher in females than in males, but the titer was not correlated with age in 118 donors (**Figures 1D–E**), although studies using all 996 donors have shown an inverse correlation between antibody titer and age (11).

### More Antigen-Specific CD8 T Cells Were Observed in Females Than in Males After the Second Vaccination

**Figure 2:**
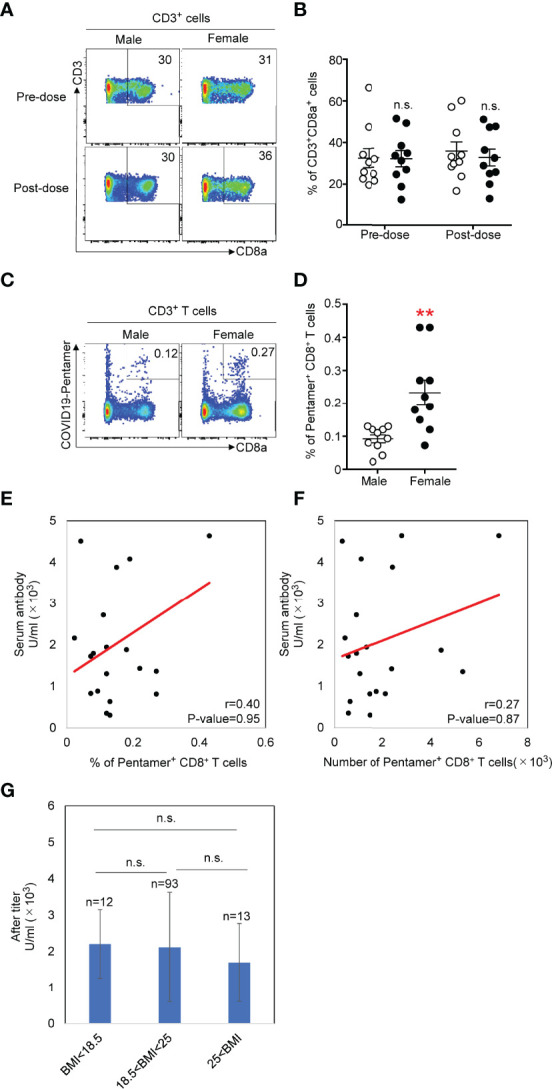
**(A)** Flow cytometric analysis of total CD8^+^ T cells isolated from PMBCs from males or females. Dot plots of CD3 and CD8 expression in propidium iodide^-^ (PI^-^) CD3^+^ PMBCs from males or females are shown. **(B)** Number of CD3^+^CD8^+^ T cells in PMBCs from males or females before and after vaccination. **(C)** Flow cytometric analysis of CD3^+^CD8^+^COVID-19-pentamer^+^ T cells isolated from PMBCs from males or females after vaccination. Plots of CD8 and COVID-19-pentamer expression in PI^-^CD3^+^ PMBCs from males or females are shown. **(D)** Number of CD3^+^CD8^+^COVID-19-pentamer^+^ T cells among PMBCs from males and females after vaccination **p < 0.01. **(E)** Scatter plots of antibody titer and COVID-19-pentamer^+^CD8^+^ T cell frequency in males and females after vaccination. **(F)** Scatter plots of antibody titer and cell number of COVID-19-pentamer^+^CD8^+^ T cells in males and females after vaccination. **(G)** Scatter plots of body mass index and cell number of COVID-19-pentamer^+^CD8^+^ T cells in males and females after vaccination. n.s., not significant.

We selected a SARS-CoV-2 S_269_ (YLQPRTFLL) pentamer to detect antigen-specific CD8 T cells in our study. This is a dominant epitope for HLA-A0201 (14), among several pentamers for HLA-A0201. Results show that this pentamer is most frequently able to detect antigen-specific T cells from COVID-19 patients (15). We tested SARS-CoV-2 S_269_ (YLQPRTFLL) pentamer^+^ cells from 20 donors randomly selected from the 118 donors (10 female and 10 male). We examined the phenotypes of SARS-CoV-2 S_269_ MHC class I pentamer^+^ CD8^+^ T cells in PBMCs one week before the 1^st^ vaccination and three weeks after the second vaccination. The total number of CD8^+^ T cells among CD3^+^ cells was not different before and after vaccination (**Figures 2A, B**). There is not pentamer^+^ cells among CD8+ T cells before vaccination in any samples, but the pentamer^+^ cells were detected 3 weeks after the second vaccination (**Figures 2C, D**). The frequency of pentamer^+^ cells 3 weeks after the 2^nd^ vaccination was significantly higher in females than in males (**Figures 2C, D**). Neither the antibody titer and frequency (**Figure 2E**) nor the number (**Figure 2F**) of pentamer^+^ cells was correlated. Furthermore, the frequency of pentamer+ cells was not correlated with the body mass index (**Figure 2G**)

### Increased Numbers of Memory Precursors of CD8+ T Cells in Females Compared With Those in Males

**Figure 3:**
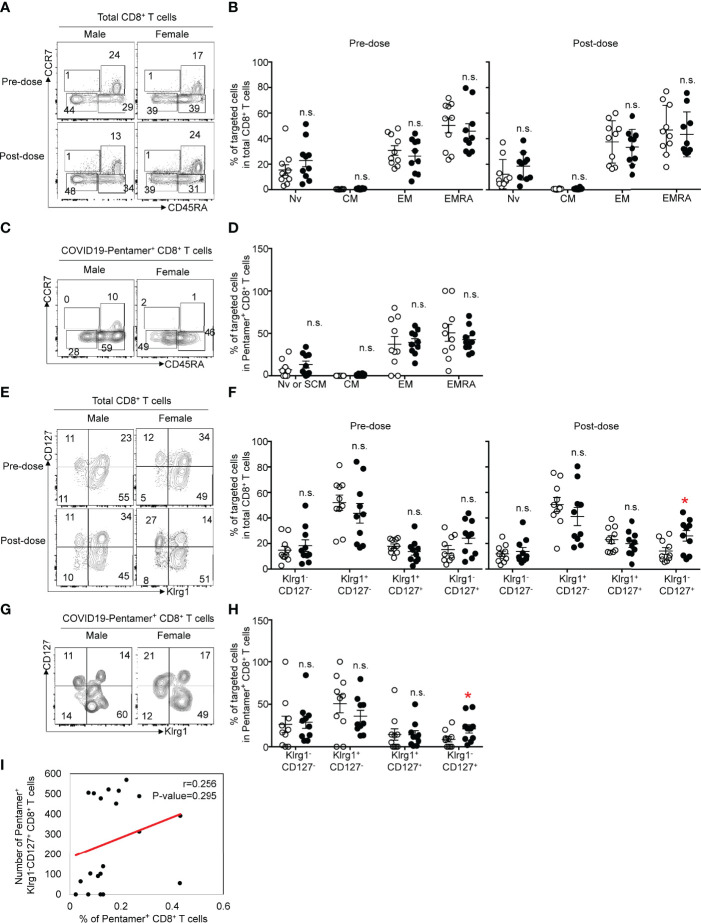
**(A)** Flow cytometric analysis of CD3^+^CD8^+^CCR7^+^CD45RA^+^ (naïve: Nv), CCR7^+^CD45RA^-^ (central memory: CM), CCR7^-^CD45RA^-^ (effector memory: EM) and CCR7^-^CD45RA^+^ (terminal effector memory: EMRA) T cells among CD8+ T cells from male or female before or after vaccination. Dot plots of CD45RA and CCR7 expression in PI^-^CD3^+^CD8^+^ PMBCs are shown. **(B)** Frequency of Nv, CM, EM and EMRA CD8^+^ T cells among PMBCs from males or females before or after vaccination. **(C)** Flow cytometric analysis of CD3^+^CD8^+^COVID-19-pentamer^+^ Nv, CM, EM and EMRA T cells among PMBCs from males or females after vaccination. Dot plots of CD45RA and CCR7 expression in PI^-^CD3^+^CD8^+^COVID-19-pentamer^+^ PMBCs are shown. **(D)** Frequency of Nv, CM, EM and EMRA COVID-19-pentamer^+^ CD8^+^ T cells among PMBCs from males or females after vaccination. **(E)** Flow cytometric analysis of CD3^+^CD8^+^KLRG1^-^CD127^-^, KLRG1^+^CD127^-^, KLRG1^+^CD127^+^ and KLRG1^-^CD127^+^ T cells among PMBCs from males or females before or after vaccination. Dot plots of KLRG1 and CD127 expression in PI^-^CD3^+^CD8^+^ PMBCs are shown. **(F)** Frequency of KLRG1^-^CD127^-^, KLRG1^+^CD127^-^, KLRG1^+^CD127^+^, and KLRG1^-^CD127^+^ CD8^+^ T cells among PMBCs from males or females before or after vaccination. **(G)** Flow cytometric analysis of CD3^+^CD8^+^COVID-19-pentamer^+^KLRG1^-^CD127^-^, KLRG1^+^CD127^-^, KLRG1^+^CD127^+^ and KLRG1^-^CD127^+^ T cells among PMBCs from males or females after vaccination. Dot plots of KLRG1 and CD127 expression in PI^-^CD3^+^CD8^+^ PMBCs are shown. **(H)** Frequency of KLRG1^-^CD127^-^, KLRG1^+^CD127^-^, KLRG1^+^CD127^+^, and KLRG1^-^CD127^+^COVID-19-pentamer^+^CD8^+^ T cells among PMBCs from males or females after vaccination. **(I)** Scatter plots of the number of pentamer^+^KLRG1^-^CD127^+^CD8^+^ T cells and the percentage of COVID-19-pentamer^+^CD8^+^ T cells after vaccination. *p < 0.05; n.s., not significant.

We next evaluated the expression patterns of cell surface markers related to effector or memory functions on pentamer^+^ CD8+ T cells. We determined similar proportions of naïve or stem-like memory T cells (CCR7^+^CD45RA^+^), central memory cells (CCR7^+^CD45RA^−^), effector memory cells (CCR7^−^CD45RA^−^) and terminally differentiated effector cells (CCR7^−^CD45RA^+^) in the CD8^+^ population between females and males before and after the second vaccination (**Figures 3A, B**). The frequency of each proportion of total CD8+ T cells after the second vaccination was equivalent to that before vaccination. We then evaluated the frequency of each population among pentamer^+^ CD8+ T cells after the second vaccination. Approximately 30% of pentamer^+^ cells exhibited an effector memory (CCR7^−^CD45RA^−^) phenotype, and ~30% or 10% of pentamer^+^ cells were terminally differentiated effector (CCR7^−^CD45RA^+^) cells or naïve/stem-like memory T cells (CCR7^+^CD45RA^+^), respectively. The frequency of those three populations in females was equivalent to that in males (**Figures 3C, D**).

The effector functions and memory precursors of CD8+ T cells are also distinguished by the expression patterns of CD127 and KLRG1 on CD8+ T cells (16). We found more KLRG1^-^CD127^+^ cells, memory precursor effector cells, among total CD8+ T cells in females than in males, but the numbers of other types of cells (KLRG1^-^CD127^-^, KLRG1^+^CD127^+^, KLRG1^+^CD127^-^) were comparable between males and females (**Figures 3E, F**). Similar to the data regarding total CD8+ T cells, the frequency of KLRG1^-^CD127^+^ cells among pentamer^+^ CD8 T cells was higher in females than in males (**Figures 3G, H**). The frequency of KLRG1^-^CD127^+^ cells among pentamer^+^ CD8+ T cells was not correlated with the frequency of pentamer^+^ cells (**Figure 3I**). These data suggest that KLRG1-CD127+ cells would be one of markers to evaluate cellular immunity after BNT162b2 mRNA vaccination.

### Increased Memory/Effector CD8+ T Cell Population in Females Compared With That in Males

**Figure 4:**
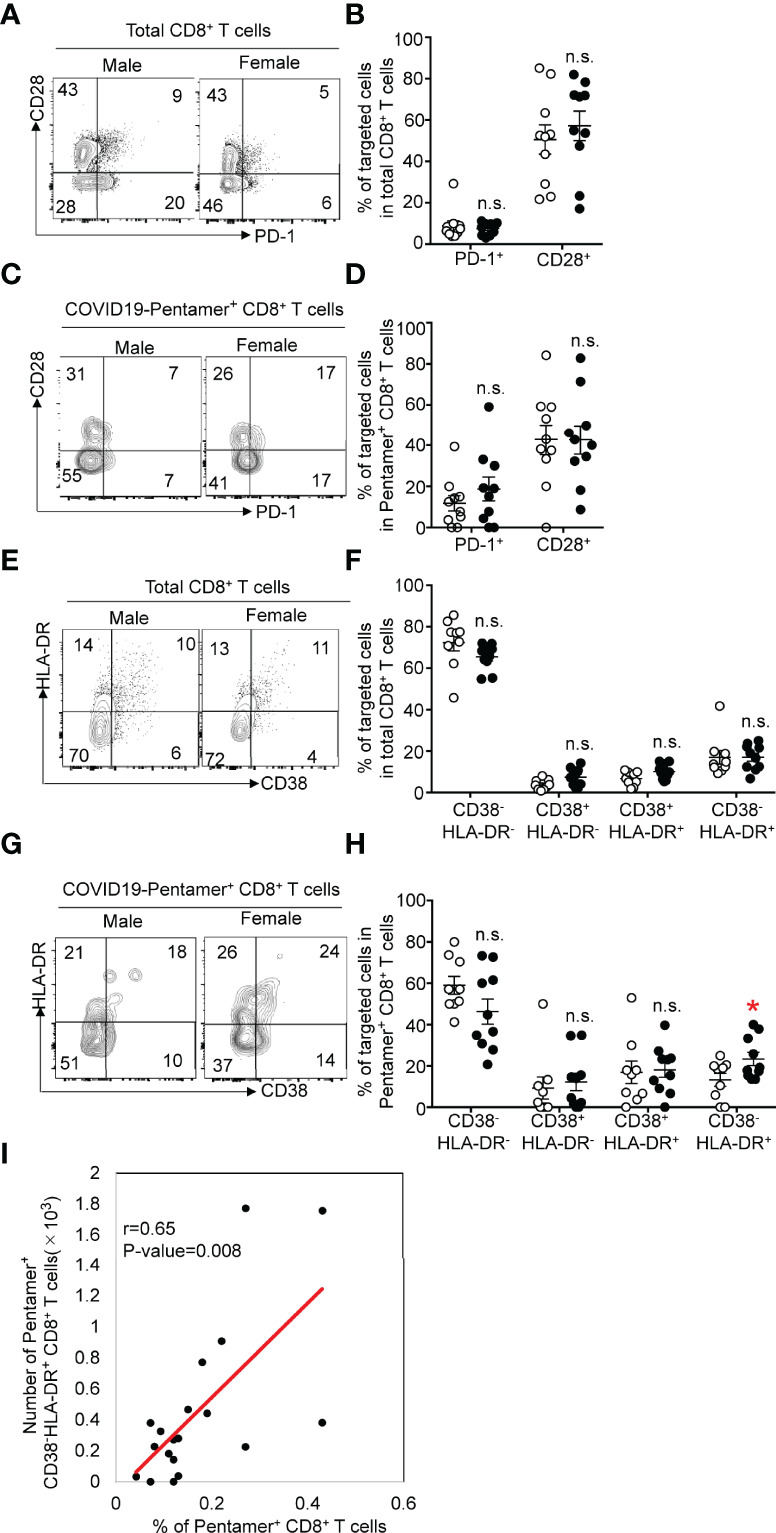
**(A)** Flow cytometric analysis of CD3^+^CD8^+^PD-1^+^ or CD28^+^ T cells among PMBCs from males or females after vaccination. Dot plots of CD28 and PD-1 expression in PI^-^CD3^+^CD8^+^ PMBCs are shown. **(B)** Frequency of CD28^+^ or PD-1^+^CD8^+^ T cells among PMBCs from males or females after vaccination. **(C)** Flow cytometric analysis of CD3^+^CD8^+^COVID-19-pentamer^+^PD-1^+^ or CD28^+^ T cells among PMBCs from males or females after vaccination. Dot plots of CD28 and PD-1 expression in PI^-^CD3^+^CD8^+^COVID-19-pentamer^+^ PMBCs are shown. **(D)** Frequency of CD28^+^ or PD-1^+^COVID-19-pentamer^+^CD8^+^ T cells among PMBCs from males or females after vaccination. **(E)** Flow cytometric analysis of CD3^+^CD8^+^CD38^-^HLA-DR^-^, CD38^+^HLA-DR^-^, CD38^+^HLA-DR^+^ and CD38^-^HLA-DR^+^ T cells among PMBCs from males or females after vaccination. Dot plots of CD38 and HLA-DR expression in PI^-^CD3^+^CD8^+^ PMBCs are shown. **(F)** Frequency of CD38^-^HLA-DR^-^, CD38^+^HLA-DR^-^, CD38^+^HLA-DR^+^ and CD38^-^HLA-DR^+^ CD8^+^ T cells among PMBCs from males or females after vaccination. **(G)** Flow cytometric analysis of CD3^+^CD8^+^COVID-19-pentamer^+^CD38^-^HLA-DR^-^, CD38^+^HLA-DR^-^, CD38^+^HLA-DR^+^ and CD38^-^HLA-DR^+^COVID-19-pentamer^+^CD8^+^ T cells among PMBCs from males or females after vaccination. Dot plots of CD38 and HLA-DR expression in PI^-^CD3^+^CD8^+^COVID-19-pentamer^+^ PMBCs are shown. **(H)** Frequency of CD38^-^HLA-DR^-^, CD38^+^HLA-DR^-^, CD38^+^HLA-DR^+^ and CD38^-^HLA-DR^+^COVID-19-pentamer^+^CD8^+^ cells among PMBCs from males or females after vaccination. **(I)** Scatter plots of the number of pentamer^+^CD38^-^HLA-DR^+^CD8^+^ T cells and percent of COVID-19-pentamer^+^CD8^+^ T cells after vaccination. *p < 0.05; n.s., not significant.

The expression of CD28 and PD-1 can be useful in distinguishing the activation status of CD8+ T cells. The frequency of CD28^+^ cells among total CD8+ T cells (**Figures 4A, B**) was equivalent to that among pentamer^+^ cells after the second vaccination (**Figures 4C, D**). The expression of PD-1 in pentamer^+^ CD8+ T cells was increased compared with that in total CD8+ T cells, and the frequency of PD-1^+^ cells among pentamer^+^ CD8+ T cells was comparable between females and males.

CD8+ T cells can also be classified by the level of activation with HLA-DR and CD38 expression patterns. Coexpression of CD38 and HLA-DR defines early activation status, with high effector functions and higher susceptibility to cell death after their function has been accomplished. CD38^-^HLA-DR^+^ CD8+ T cells exhibit an elevated functional response with an increased survival rate during viral infection (17). Four populations based on the expression pattern of CD38 and HLA-DR were detected in pentamer^+^ cells. Although the frequency of CD38^+^HLA-DR^+^ or CD38^-^HLA-DR^+^ cells among total CD8+ T cells was comparable between females and males (**Figures 4E, F**), we found a similar frequency of CD38^+^HLA-DR^+^ cells in females and males and a higher frequency of CD38^-^HLA-DR^+^ CD8+ T cells among pentamer^+^ CD8+ T cells in females than in males (**Figures 4G, H**). The number of CD38^-^HLA-DR^+^ cells among pentamer^+^ CD8+ T cells was correlated with the frequency of pentamer^+^ cells (**Figure 4I**). These data suggest that females are able to generate higher functional responses by the second dose of BNT162b2.

### Memory T Cell Responses

**Figure 5:**
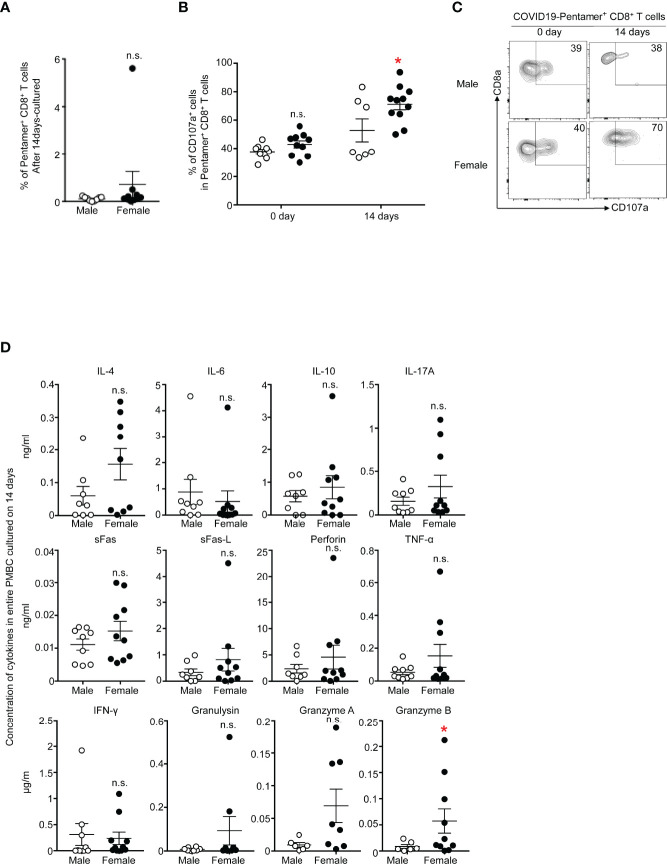
**(A)** Frequency of PI-CD3+CD8+COVID-19-pentamer+ PMBCs from males and females cultured for 14 days. **(B)** Frequency of CD107a^+^COVID-19-pentamer^+^CD8^+^ T cells among PMBCs from males and females cultured for 0 days and 14 days. **(C)** Flow cytometric analysis of CD107a^+^COVID-19-pentamer^+^CD8^+^ T cells among PMBCs from males and females cultured for 0 days and 14 days. Dot plots of CD8 and CD107a expression in PI^-^CD3^+^CD8^+^COVID-19-pentamer^+^ PMBCs are shown. **(D)** LEGENDplex™ multianalyte flow assay of IL-4, IL-6, IL-10, IL-17A, sFas, sFas-L, perforin, TNF-α, IFNγ, granulysin, granzyme A and granzyme B from PMBCs from males and females cultured for 0 days and 14 days. *p < 0.05; n.s., not significant.

We next tested the epitope-specific effector functions of CD8+ T cells by stimulating PBMCs with an S protein-derived peptide for 14 days. We tested the frequency of antigen-specific T cells, cytotoxic functions and cytokine secretion levels. The stimulation of T cells with the peptides led to the expansion of T cells, and the frequency of pentamer^+^CD8+ T cells tends to be greater in females than in males (**Figure 5A**). The killing capability of pentamer^+^CD8+ T cells was assessed by staining cell surface CD107a, a marker for degranulation, before and after T cell stimulation. Approximately 40% of pentamer^+^ T cells expressed CD107a before stimulation, and the frequency was similar between males and females. Upon stimulation with the peptide, more than 70% of pentamer^+^ cells expressed CD107a, and the frequency was higher in females than in males (**Figure 5B**). The intensity of CD107a staining tended to be higher in females than in males (**Figure 5C**).

The concentrations of molecules secreted from T cells were measured 14 days after T cell stimulation. IFN-γ and TNF-α tended to be more highly secreted in cells from females than in cells from males, but the levels were comparable between females and males (**Figure 5D**). Only granzyme B was secreted from T cells in greater amounts by females than by males in this study, suggesting stronger cytotoxic responses in females than males during the memory phase after the second COVID-19 vaccination.

## Discussion

The adaptive immune system is essential for preventing or clearing the virus in the body (18–21). Vaccines against virus infection activate both innate and adaptive immunity by mimicking natural infections (18, 22). Antibodies are crucial for preventing the entry of free virus into cells, and the vaccine effect is easily determined by measuring the antibody titer in the serum. In contrast, although T cells are needed to clear infected cells, the measurement of antigen-specific T cells is not easy because HLA typing and determination of the antigen epitope is required to detect the T cells. Here, we investigated the frequency, cell surface markers and function of antigen-specific CD8 T cells from donors who received a second BNT162b2 mRNA vaccine. In the present study, we demonstrated that SARS-CoV-2-specific memory T cell responses were more abundant in females than in males. Although several paper reported the generation of stable memory T cells after BNT162b2 mRNA vaccination (23, 24), those studies did not show which cell surface markers are useful to assess the vaccine effect on cellular immunity. In that sense, we here found that CD127^+^KLRG1^-^ cells was more abundant in females than in males. This study suggests that CD127^+^KLRG1^-^ cells could be used as one of indicators of memory CD8+ T cell generation by BNT162b2 vaccination although it needs to be analyzed with larger number of donors and a variety of age groups in the future.

Our previous study and other studies showed a higher titer of anti-S protein antibodies in females than in males (11, 25). Other studies reported that a higher risk of certain types of adverse events were observed among women (26). In this study, we detected a higher number of antigen-specific CD8+ T cells in females than in males. How can the sex difference in the immune response against BNT162b2 mRNA vaccine in the Japanese population be explained? The involvement of body weight is unlikely because we did not observe a positive correlation between BMI and the frequency of antigen-specific T cells or antibody titer in this study (11). The most likely explanation is distinct immune responses against the vaccine between females and males, at least in the Japanese population. Previous studies have also shown stronger immune responses in females than in males after vaccination against viruses, including influenza virus (27). The sex hormone might be responsible for the difference. For instance, men with high testosterone levels mounted lower titers of neutralizing antibody suggesting an immunosuppressive role for testosterone in the context of influenza vaccination (28). It is also noted that there is low and high responder against the vaccine or peptide-mediated stimulation *in vitro* even in females. Although we do not have information about menstrual cycle in our donors, the hormone cycle might be involved in the T cell responses even in females. Future studies on detailed molecular signatures in pentamer^+^ T cells in female and male donors could clarify the differential cellular immune responses against BNT162b2 vaccination. In addition, all donors in this study were healthcare workers who had been involved in the care of COVID-19 patients. It is unlikely that T cells from donors involved in the care of COVID-19 patients were suboptimally primed because donors in this study showed levels of anti-S antibody and antigen-specific T cells that were under the limits of detection before vaccination.

By testing several cell surface markers that distinguish subsets of memory and effector T cells, we detected an increased frequency of CD8^+^KLRG1^-^CD127^+^ cells in females compared with that in males. The same tendency was observed for pentamer+ cells. As KLRG1^-^CD127^+^ cells are defined as memory precursor effector cells that display an increased ability to form long-lived memory cells and short-lived effector cells (29), female donors should have better potential to generate memory CD8+ T cells. As we still do not have many COVID-19 cases even after vaccination, we are not able to assess whether female donors are more resistant to SARS-CoV-2 after a second dose of vaccination. Nevertheless, upon *in vitro* stimulation of CD8 T cells with peptides, there was higher expression of cytotoxicity-related molecules in female donors than in male donors, suggesting stronger memory T cell responses in females than in males after the second vaccination. In future studies, it will be helpful to directly compare the function of pentamer+KLRG1^-^CD127^+^ cells and CD8^+^KLRG1^-^CD127^+^ cells between female and male subjects.

CD38^+^HLA-DR^+^pentamer^+^ T cells were detected 21 days after the second vaccination, and the frequency was higher in females than in males, suggesting that this population would also be an indicator for the generation of memory CD8+ T cells. Regarding the distinct expression patterns of CD38 and HLA-DR between natural infection and vaccination and since impairment of dendritic functions in patients with COVID-19 has been reported (30), the lack of impairment of dendritic cells might lead to strong stimulation of CD8+ T cells by vaccination.

Polyclonal T cells are crucial for clearing virus infection, and the generation of polyfunctional memory T cells by vaccination is required for conferring protective immunity. Here, we tested memory CD8+ T cells against only one epitope on HLA-A*0201. Therefore, it would be necessary to evaluate the frequency and functions of CD8+ T cells across a broad range of HLA class I alleles and SARS-CoV-2 peptides to understand a more detailed landscape of CD8+ T cell responses in COVID-19 in future studies. However, even when evaluating total CD8+ T cells, we detected increased numbers of KLRG1^-^CD127^+^ cells in females compared with those in males, strongly suggesting that CD8^+^KLRG1^-^CD127^+^ cells would be a useful indicator for evaluating the generation of memory CD8+ T cells by BNT162b2 mRNA vaccination without using pentamer staining.

## Data Availability Statement

The raw data supporting the conclusions of this article will be made available by the authors, without undue reservation.

## Ethics Statement

The studies involving human participants were reviewed and approved by the Human Research Committee in Chiba University and Human Research Committee in Tokushima University. The patients/participants provided their written informed consent to participate in this study.

## Author Contributions

HK, HN, and KY designed the studies. HK performed all experiments. KO and S-IT analyzed the data. TK, ST, YM, and YO collected the samples. HK and KY wrote the papers. HN and KY supervised all researches. All authors contributed to the article and approved the submitted version.

## Funding

This study is supported by JST Moonshot R&D grant numbers JPMJMS2025 and a funding from Tokushima University - Technion - Nichia Corporation collaboration. Nichia Corporation was not involved in the study design, collection, analysis, interpretation of data, the writing of this article or the decision to submit it for publication. 

## Conflict of Interest

The authors declare that the research was conducted in the absence of any commercial or financial relationships that could be construed as a potential conflict of interest.

## Publisher’s Note

All claims expressed in this article are solely those of the authors and do not necessarily represent those of their affiliated organizations, or those of the publisher, the editors and the reviewers. Any product that may be evaluated in this article, or claim that may be made by its manufacturer, is not guaranteed or endorsed by the publisher.
